# Supplementary material for: Magnetic field alignment of stable proton-conducting channels in an electrolyte membrane
Source: Nat Commun. 2019 Feb 19;10:842. doi: 10.1038/s41467-019-08622-2 (PMC6381100; doi:10.1038/s41467-019-08622-2)
Supplement: Supplementary file 1 — Supplementary Information [file 41467_2019_8622_MOESM1_ESM.pdf]

Supplementary Information

for

Magnetic field alignment of stable proton-conducting channels in an electrolyte membrane

Liu et al.

Supplementary Note 1. Characterization of CP4VP

X-Ray photoelectron spectroscopy (XPS) measurements shown Supplementary Figure 1a are first carried out to confirm the CP4VP synthesis. C 1s and N 1s peaks at 285 eV and 533 eV, respectively, are observed for P4VP, and the atomic ratio of C/N is 7.13, which is very close to the theoretical value. For the target CP4VP, the most evident difference is the appearance of peaks at 708 eV and 721 eV, which correspond to the $2p_{3/2}$ and $2p_{1/2}$ signals for Fe, respectively, verifying that the ferrocyanide coordinating group is linked to P4VP. The atomic ratio of C/N in CP4VP becomes 2.02, much lower than that of P4VP, which is ascribed to the addition of multiple cyano groups in the coordinating group. The comparative FTIR spectra in Supplementary Figure 1b show absorption bands at 1595 cm^{-1}, 1557 cm^{-1} and 1414 cm^{-1} for P4VP, which are ascribed to the $v_{C=N}$, $v_{C=C}$ and $v_{C\text{-}N}$ of the pyridine rings, respectively[1, 2]. The CP4VP polymer is distinguished by a very strong sharp characteristic band at 2046 cm^{-1} corresponding to $v_{C\equiv N}$, which verifies the reaction of P4VP with SPCAF[3]. Moreover, all the absorptions in pyridine rings mentioned above show slight red shifts to 1603 cm^{-1}, 1560 cm^{-1} and 1420 cm^{-1}, respectively, indicating coordination of the pyridine rings[1, 2]. The TGA results shown in Supplementary Figure 1c further confirm the synthesis. The initial weight loss of P4VP occurring in the range of 30-100 ^{o}C is related with water loss, and the second weight loss between 330-430 ^{o}C corresponds to the complete decomposition of P4VP[4]. The initial water loss for CP4VP occurs over a wider range of 30-200 ^{o}C, because of the hydrophilicity afforded by the coordinating groups. A second degradation step at about 300 ^{o}C

occurs from the loss of C≡N groups in CP4VP[5]. Different from P4VP, the CP4VP has a char residue of 23.8% from the non-volatile Fe content. Supplementary Figure 1d shows the comparative DSC curves after elimination of thermal history by quenching. A higher T_g for CP4VP indicates less chain mobility from additional steric hindrance of the coordinating groups and dipolar interactions. Here it is worth noting that ^1H NMR data for CP4VP could not be obtained because of its insolubility in any deuterated organic solvent. While CP4VP is soluble in D_2O, the active protons would become deuterated, thus leading to an unreliable result.

The ion exchange capacity (IEC) of CP4VP is investigated only by standard titration, since evaluation by ^1H NMR was not practical. The experimental IEC value of CP4VP is 10.22 mmol g^{-1}, which is very close to the theoretical value of 10.20 mmol g^{-1} (Supplementary Table 1). This indicates that all the P4VP pyridyl groups react with SPCAF and the CP4VP is completely acidified after the treatment with 1 M HCl. A similar conclusion is also obtained by elemental analysis. Supplementary Table 1 shows that the experimental and theoretical values of H, C and N in CP4VP are almost identical, indicating complete conversion for both the coordination of P4VP and the acidification of CP4VP.

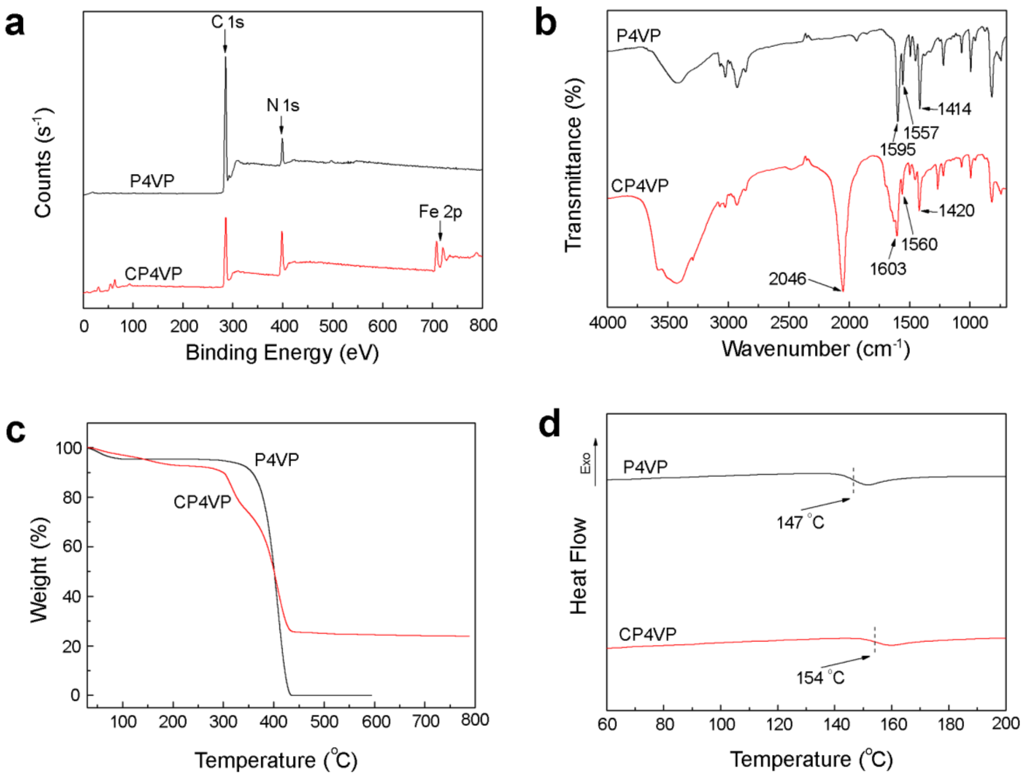

Supplementary Figure 1. Comparative characterization of P4VP and CP4VP supporting the reaction between P4VP and SPCAF to produce CP4VP. **a** XPS. **b** FTIR, KBr pellets. **c** TGA. **d** DSC. Source data are provided as a Source Data file.

Supplementary Table 1. IEC titration values and elemental analysis of CP4VP

CP4VP	IEC (mmol g^{-1})	H (wt%)	C (wt%)	N (wt%)
Experimental value	10.22 ±0.09	3.46 ±0.17	48.99 ±1.23	28.51 ±0.91
Theoretical value[a]	10.20	3.40	48.98	28.57

[a] Calculations are according to the structure of the fully acidified repeat unit in CP4VP,

that is, 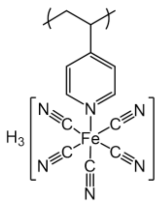, with the stoichiometry of $H_{10}C_{12}N_6Fe$.

Supplementary Table 2. Values of magnetic susceptibility (χ) for starting materials and membranes at 80 ^{o}C

Material or membrane	χ^{a} ($\times10^{-6}$)
CP4VP	-2.46
PWA	-0.62
PSf	-3.43
NM-45PC-TP	-2.63
NM-45PC-IP	-2.72
MM-45PC-TP	21.1
MM-45PC-IP	3.76

[a] χ is calculated by the following equation:

$$\chi = \frac{M}{H}$$

where M is the magnetization of the sample, and H is the magnetic field intensity.

Supplementary Note 2. Membrane electron conductivity

Membrane proton conductivity is conventionally obtained by the AC impedance method, because the electron conductivity of PEM is usually negligible. The conductivity calculated from AC impedance measurement is the total conductivity, i.e. the sum of proton conductivity (highly RH dependent) and electron conductivity (RH independent)[6]. Since MM-45PC is a novel PEM with the possibility of having a significant electron conductivity, it was investigated to ensure its feasibility in PEMFC application.

Materials based on heteropoly blue have been reported to be electron conductive, but their electron conductivities differed over quite a wide range. Some materials[7] had high electron conductivities up to the level of 10^{-2} S cm^{-1}, while others[8] had very low overall combined conductivity of 10^{-11} S cm^{-1} under 45% RH (using AC impedance), indicating the electron conductivity was even lower.

The membrane TP and IP electron conductivities of MM-45PC are measured by the DC resistance method, and are in the range of 10^{-7} ~ 10^{-8} S cm^{-1}, which is much lower than those of Nafion® 212 (10^{-5} ~ 10^{-6} S cm^{-1}), as shown in Supplementary Figure 2. Thus, the relative contribution of electron conductivity of MM-45PC toward the combined proton and electron conductivities obtained by AC impedance (Fig. 9a) is negligible.

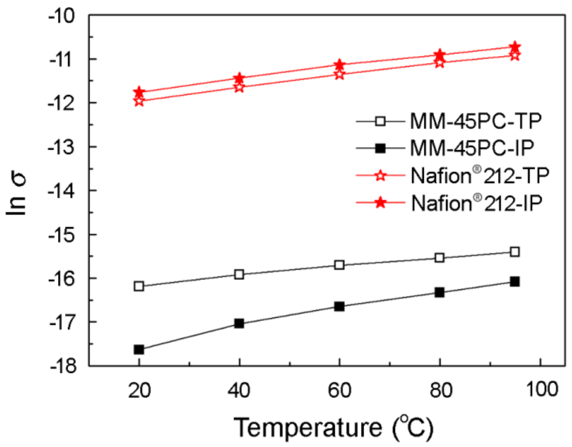

Supplementary Figure 2. Comparative electron conductivities of MM-45PC and Nafion® 212 measured in water at various temperatures using the DC resistance method. Both TP and IP electron conductivities of MM-45PC are much lower than those of Nafion® 212. Source data are provided as a Source Data file.

Supplementary Table 3. Activation energies (E_as) for the proton conductivity of MM-45PC and Nafion® 212 in water

Membrane	E_a in TP (kJ mol^{-1})	E_a in IP (kJ mol^{-1})
MM-45PC	8.70	14.93
Nafion® 212	12.29	12.19

Supplementary Table 4. IEC of MM-45PC before and after stability test

MM-45PC	Titration value before stability test	Titration value after stability test	Theoretical value[a]
IEC (mmol g^{-1})	1.48 ±0.04	1.51 ±0.03	1.50

[a] Calculation is according to the original materials in MM-45PC, that is,

$$\mathrm{IEC_{MM\text{-}45PC}} = \mathrm{IEC_{CP4VP}} \cdot \mathrm{Loading_{CP4VP}} + \mathrm{IEC_{PWA}} \cdot \mathrm{Loading_{PWA}} = 10.20\ \mathrm{mmol\ g^{-1}} \times 11.25\ \mathrm{wt\%} + 1.04\ \mathrm{mmol\ g^{-1}} \times 33.75\ \mathrm{wt\%} = 1.50\ \mathrm{mmol\ g^{-1}}$$

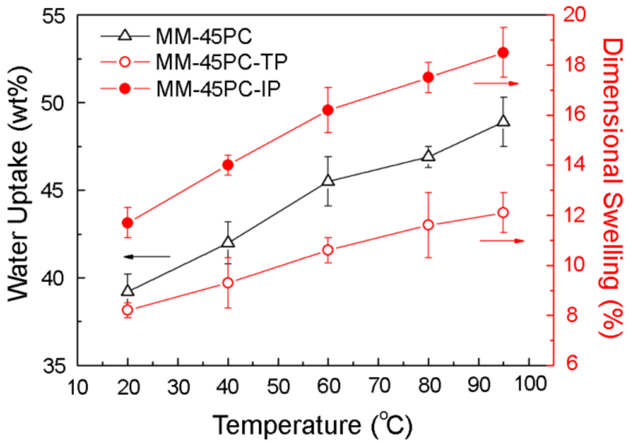

Supplementary Figure 3. Water uptake and dimensional swelling of MM-45PC measured in water. Both water uptake and dimensional swelling are in a useful range for PEMs at various temperatures (standard deviation type error bar). Source data are provided as a Source Data file.

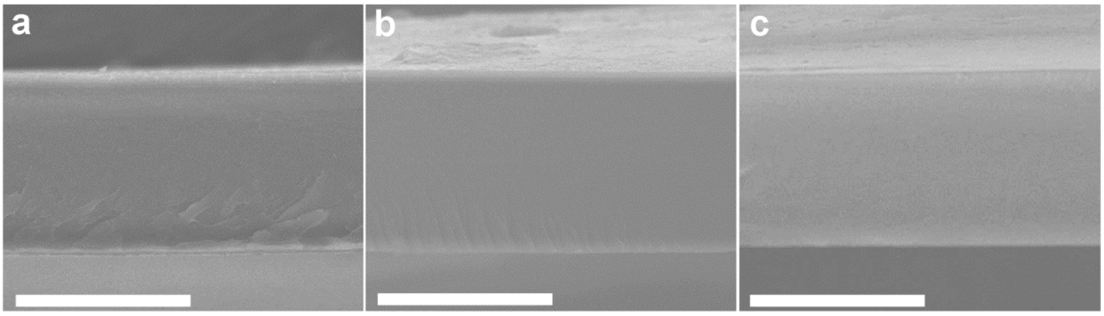

Supplementary Figure 4. Cross-sectional SEM images of MM-45PC membrane samples used for in situ PEMFC tests. **a** Sample 1 (53 μm) used for the 95 ^{o}C/100% RH polarization curve and constant voltage durability test (50 μm scale bar). **b** Sample 2 (54 μm) used for the chemical AST (50 μm scale bar). **c** Sample 3 (51 μm) used for the mechanical AST (50 μm scale bar). All samples have very similar thickness in the range of 51 to 54 μm, very close to the 50.8 μm of Nafion® 212, which ensures data comparability in the in situ PEMFC test. The cross-sectional SEM images also reveal that each MM-45PC sample has uniform thickness.

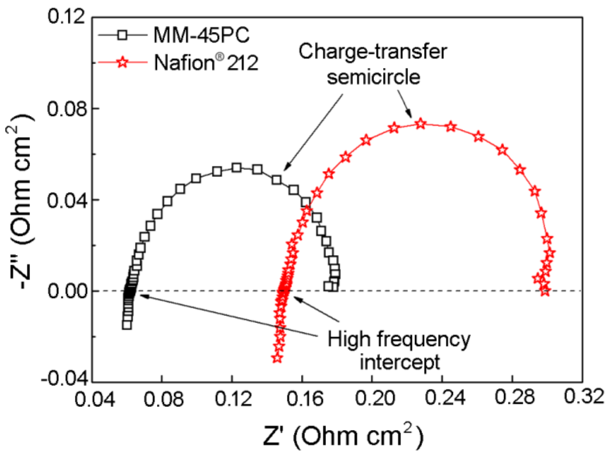

Supplementary Figure 5. Comparative in situ EIS analyses of the PEMFCs based on MM-45PC (0.45 V, 2459 mA cm^{-2}) and Nafion® 212 (0.45 V, 1431 mA cm^{-2}) at their power density maxima. There is a marked difference (140%) in their high frequency intercepts, indicating the in situ membrane resistance of MM-45PC is considerable lower than that of Nafion® 212, which is primarily responsible for the higher power density of MM-45PC. Although it appears contradictary that there is only a small difference (18%) in the ex situ TP conductivity (Fig. 9a) and such a substantial difference (140%) in the in situ membrane resistance, it must be emphasized that the proton conductivity data in Fig. 9a were tested at 95 ^{o}C in liquid water, while the PEMFC data were at 95 ^{o}C at 100 %RH. When TP proton conductivity was measured under the same conditions used in the PEMFC test, at 95 ^{o}C and 100% RH, a large difference (89%) between MM-45PC and Nafion® 212 is observed. The difference in membrane proton conductivities between a liquid water environment and 100% RH atmosphere are quite marked for Nafion® 212, which is one of the principal reasons for exploring other PEMs. The perfluorinated structure of Nafion® 212 is susceptible to severe membrane dehydration under water vapor atmosphere at an elevated

temperature of 95 ^{o}C, without the osmotic pressure of liquid water. Moreover, the PEMFC current density of MM-45PC is much greater than that of Nafion® 212 in EIS tests, so the faster water generation incurs extra humidification for MM-45PC, which further enhances the increase to 140%. Since the current density of MM-45PC PEMFC is larger than that of Nafion® 212 PEMFC, MM-45PC PEMFC also exhibits a charge-transfer semicircle with a smaller diameter (33% change) due to the reduced charge-transfer resistance, which is a common phenomenon for the same catalyst system. The difference in charge-transfer resistance (33%) is much lower than that in membrane resistance (140%) (dashed lines are used to guide the eyes). Source data are provided as a Source Data file.

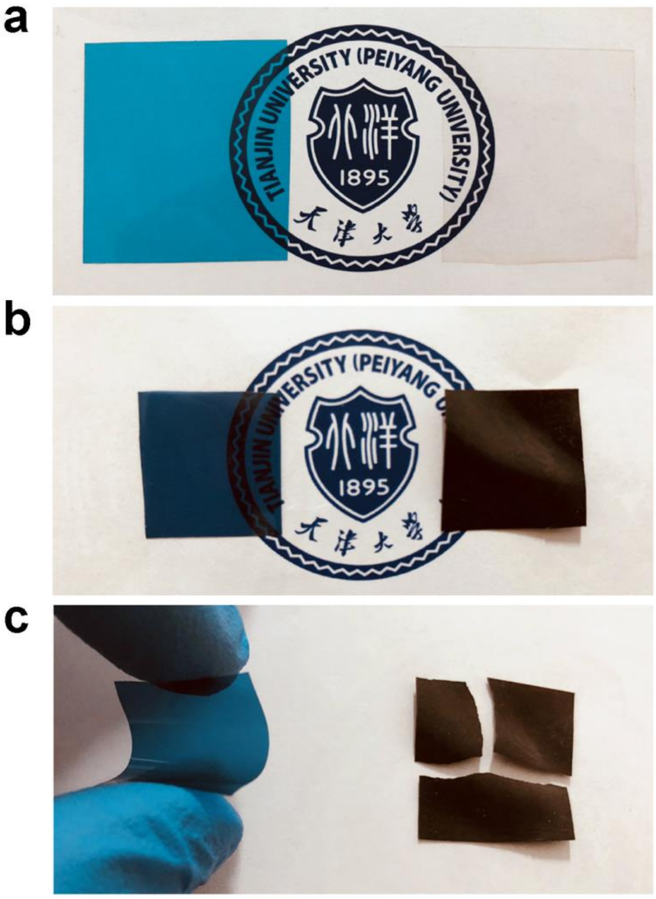

Supplementary Figure 6. Visual appearance of MM-45PC (left in each subfigure) and Nafion® 212 (right in each subfigure) before and after PEMFC durability tests. **a** Before durability tests, both membranes are homogeneous and transparent. The membranes are about 3×3 cm^2. **b** After durability tests, the effective membrane areas of 2×2 cm^2 are cut from the original size. The membranes are treated with five cycles of liquid helium soaking/water ultrasonic bath to remove most of the catalyst layer. MM-45PC retains a blue color but darker, which is likely due to the ingress of small amounts of catalyst, but it is still homogeneous and transparent. In contrast, Nafion® 212 has a black-brown coloration with an uneven and completely opaque surface, indicating severe degradation. **c** After durability tests, MM-45PC maintains flexibility, but Nafion® 212 membrane is brittle and readily fractures when bent.

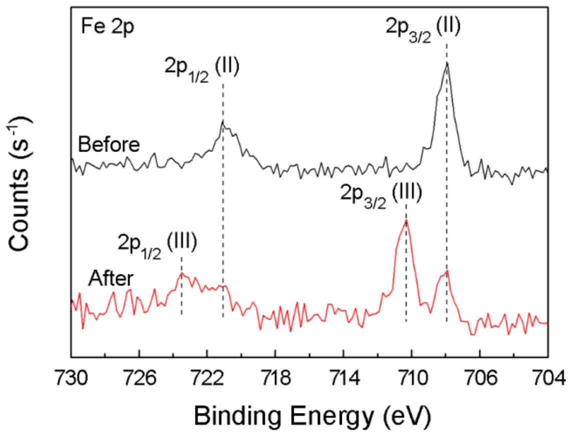

Supplementary Figure 7. XPS narrow scans for Fe in MM-45PC before and after chemical AST (90 ^{o}C/30% RH OCV hold with H_2/O_2). Before chemical AST, the MM-45PC membrane displays only the signal of Fe (II), corresponding to ferrocyanide. After chemical AST, both Fe (II) and Fe (III) signals appear, indicating a redox reaction of ferrocyanide and ferricyanide occurs during OCV hold. The coexistence of comparable amounts of ferrocyanide and ferricyanide after long-term OCV hold reflects that the redox reaction is in equilibrium with rapid interconversions between Fe (II) and Fe (III), so reactions with free radicals are continuously renewable, which helps to mitigate chemical degradation of the PEM. Source data are provided as a Source Data file.

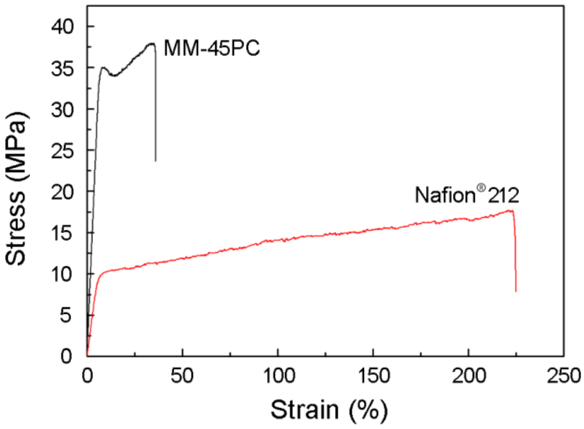

Supplementary Figure 8. Stress-strain curves of MM-45PC and Nafion® 212. Compared with Nafion® 212, the MM-45PC has a much higher elastic modulus (initial slope) and tensile strength of MM-45PC. The breaking elongation of Nafion® 212 is higher than that of MM-45PC. Source data are provided as a Source Data file.

Supplementary Table 5. Comparison with related research

Ref.	Focus	Filler	Method	Fuel cell output	Filler retention	In situ stability
9	Alignment	Palladium	Electric field	30 mW cm^{-2} at 40 ^{o}C with methanol/O_2	-	-
10	Alignment	Fe_2O_3	Magnetic field	92.4 mW cm^{-2} at 70 ^{o}C with methanol/O_2	-	-
11	Alignment	Chitosan coated iron oxide	Magnetic field	455 mW cm^{-2} at 120 ^{o}C under 100% RH with H_2/O_2	-	-
12	Filler retention	PWA	Embedded in meso-silica	308 mW cm^{-2} at 80 ^{o}C under 80% RH with H_2/O_2	28% loss in proton conductivity within 6 h	-
13	Filler retention	PWA	Embedded in nanotube	573 mW cm^{-2} at 120 ^{o}C under 100% RH with H_2/O_2	No PWA leaching within 250 h at 120 ^{o}C and 40% RH	-
14	Filler retention	PWA	Electrostatic force with polymer	618 mW cm^{-2} at 50 ^{o}C under 100% RH with H_2/O_2	No PWA leaching within 500 h in 20 ^{o}C water	-
15	In situ stability	MnO_2	Simple incorporation	1200 mW cm^{-2} at 80 ^{o}C under 100% RH with H_2/O_2 (0.3 MPa pressure)	-	2500 $\mu V\ h^{-1}$ loss in 120 h OCV hold at 80 ^{o}C under 50% RH with H_2/O_2
16	In situ stability	Vitamin E	Simple incorporation	~ 600 mW cm^{-2} at 65 ^{o}C under 100% RH with H_2/O_2	-	~1000 $\mu V\ h^{-1}$ loss in 120 h OCV hold at 65 ^{o}C under 100% RH with H_2/O_2

17	In situ stability	Dihydroxy -cinnamic acid	Simple incorporation	Less than 100 mW cm^{-2} at 90 ^oC under 30% RH with H_2/O_2	-	7.27 % power density loss within 120 h at 90 ^oC under 30% RH with H_2/O_2
18	Filler retention	Lacunary SiWA	Tethered with polymer	1140 mW cm^{-2} at 80 ^oC under 100% RH with H_2/O_2	Obvious decrease in proton conductivity after 16 h in 80 ^oC water	100 $\mu V\ h^{-1}$ loss in 500 h OCV hold at 90 ^oC under 30% RH with H_2/O_2
	In situ stability	Lacunary SiWA	Radical decomposition catalyst			
This work	Alignment	CP4VP /PWA	Magnetic field	1107 mW cm^{-2} at 95 ^oC under 100% RH with H_2/O_2	No PWA leakage within 30 days (720 h) in 95 ^oC water	13 $\mu V\ h^{-1}$ loss in OCV hold at 90 ^oC under 30% RH with H_2/O_2, 1.0% OCV loss within 32 days (768 h)
	Filler retention	PWA	Tethered with proton conductive polymer			
	In situ stability	CP4VP	Radical scavenging by negatively charged redox group on polymer			

Supplementary References

1. Fan, S. et al. Monodispersed poly(4-vinylpyridine) spheres supported Fe(III) material: an efficient and reusable catalyst for benzylic oxidation. *J. Mol. Catal. A: Chem.* **404-405**, 186-192 (2015).
2. Wu, K. H., Wang, Y. R., & Hwu, W. H. FTIR and TGA studies of poly(4-vinylpyridine-co-divinylbenzene)–Cu(II) complex. *Polym. Degrad. Stab.* **79**, 195-200 (2003).
3. Liu, Y., & Wang, X. Synthesis, characterization, micellization and metal coordination polymerization of pentacyanoferrate-coordinated block copolymers for monodispersed soluble Prussian blue nanospheres. *Polym. Chem.* **3**, 2632-2639 (2012).
4. Kollmetz, T., Georgopanos, P., & Handge, U. A. Rheology in shear and elongation and dielectric spectroscopy of polystyrene-block-poly(4-vinylpyridine) diblock copolymers. *Polymer* **129**, 68-82 (2017).
5. Gerber, S. J., & Erasmus, E. Electronic effects of metal hexacyanoferrates: an XPS and FTIR study. *Mater. Chem. Phys.* **203**, 73-81 (2018).
6. Hatakeyama, K. et al. Tunable graphene oxide proton/electron mixed conductor that functions at room temperature. *Chem. Mater.* **26**, 5598-5604 (2014).
7. Ouahab, L. et al. Preparations, X-ray crystal structures, EH band calculations, and physical properties of [$(TTF)_6(H)(XM_{12}O_{40})(Et_4N)$] (M = tungsten,

molybdenum; X = phosphorus, silicon): evidence of electron transfer between organic donors and polyoxometalates. *Chem. Mater.* **4**, 666-674 (1992).

8. Li, C., Sun, M., Xu, L., Wang, Y., & Huang, J. The first heteropoly blue-embedded metal–organic framework: crystal structure, magnetic property and proton conductivity. *CrystEngComm* **18**, 596-600 (2016).

9. Zhang, X., Zhang, Y., Nie, L., Liu, X., & Chang, L. Modification of Nafion membrane by Pd-impregnation via electric field. *J. Power Sources.* **216**, 526-529 (2012).

10. Chang, C.-M., Li, H.-Y., Lai, J.-Y., & Liu, Y.-L. Nanocomposite membranes of Nafion and Fe_3O_4-anchored and Nafion-functionalized multiwalled carbon nanotubes exhibiting high proton conductivity and low methanol permeability for direct methanol fuel cells. *RSC Adv.* **3**, 12895-12904 (2013).

11. Hasani-Sadrabadi, M. M. et al. Magnetically aligned nanodomains: application in high-performance ion conductive membranes. *ACS Appl. Mater. Interfaces* **6**, 7099-7107 (2014).

12. Zhou, Y. et al. Insight into proton transfer in phosphotungstic acid functionalized mesoporous silica-based proton exchange membrane fuel cells. *J. Am. Chem. Soc.* **136**, 4954-4964 (2014).

13. Hasani-Sadrabadi, M. M. et al. Ionic nanopeapods: next-generation proton conducting membranes based on phosphotungstic acid filled carbon nanotube. *Nano Energy* **23**, 114-121 (2016).

14. Lu, S. et al. A self-anchored phosphotungstic acid hybrid proton exchange

membrane achieved via one-step synthesis. *Adv. Energy Mater.* **4**, 1400842 (2014).

15. Zhao, D., Yi, B. L., Zhang, H. M., & Yu, H. M. $MnO_2/SiO_2–SO_3H$ nanocomposite as hydrogen peroxide scavenger for durability improvement in proton exchange membranes. *J. Membr. Sci.* **346**, 143-151 (2010).

16. Yao, Y. et al. Vitamin E assisted polymer electrolyte fuel cells. *Energy Environ. Sci.* **7**, 3362-3370 (2014).

17. Park, Y., & Kim, D. Chemical stability enhancement of Nafion membrane by impregnation of a novel organic ·OH radical scavenger, 3,4-dihydroxy-cinnamic acid. *J. Membr. Sci.* **566**, 1-7 (2018).

18. Motz, A. R. et al. Heteropoly acid functionalized fluoroelastomer with outstanding chemical durability and performance for vehicular fuel cells. *Energy Environ. Sci.* **11**, 1499-1509 (2018).
